# Comparative Effectiveness of Prophylactic Vasopressors During Elective Cesarean Delivery Under Neuraxial Anesthesia: A Systematic Review With Network-Based Quantitative Synthesis

**DOI:** 10.7759/cureus.101603

**Published:** 2026-01-15

**Authors:** Arya Babul, Sohi Ashraf, Leanne Free, Jyoti Desai, Momina Hussain, Najib Babul

**Affiliations:** 1 Biomedical Sciences, West Career and Technical Academy, Society for Awareness of Neglected Diseases, Las Vegas, USA; 2 Critical Care Medicine, MountainView Hospital, Las Vegas, USA; 3 Gynecologic Surgery and Obstetrics, Kirk Kerkorian School of Medicine at the University of Nevada, Las Vegas, Las Vegas, USA; 4 Genomics, Chinese Academy of Tropical Agricultural Sciences, Sanya, CHN; 5 Drug Development, Quadra Therapeutics, Las Vegas, USA

**Keywords:** anesthesia spinal, cesarean section (cs), ephedrine, low-risk elective cesarean delivery, network meta-analysis, neuraxial anesthesia complications, norepinephrine, phenylephrine, postspinal hypotension

## Abstract

Cesarean delivery is commonly performed under spinal anesthesia because of its rapid onset, effective analgesia, and favorable maternal-fetal safety profile. Postspinal hypotension, however, remains a frequent complication and may result in maternal symptoms and impaired uteroplacental perfusion. Vasopressors such as phenylephrine, norepinephrine, and ephedrine are routinely used for prophylaxis, yet the optimal agent in low-risk parturients remains uncertain. This study compared the effectiveness and safety of commonly used prophylactic vasopressors in low-risk women undergoing elective cesarean delivery under spinal anesthesia. PubMed, Embase, CENTRAL, and ClinicalTrials.gov were systematically searched from inception to May 2025, and the protocol was registered on PROSPERO (CRD420251060720). Randomized controlled trials comparing norepinephrine, phenylephrine, ephedrine, or placebo were included. In total, 42 trials involving 4,534 participants met the eligibility criteria. A network meta-analysis using odds ratios with 95% confidence intervals was conducted, and treatments were ranked using the surface under the cumulative ranking curve values. Compared with placebo, vasopressors showed a non-significant trend toward preventing postspinal hypotension. Norepinephrine ranked highest for favorable maternal hemodynamic outcomes and prevention of bradycardia, followed by phenylephrine and ephedrine. No significant differences were observed in neonatal Apgar scores or maternal nausea and vomiting. Overall, while statistical superiority was not demonstrated, norepinephrine consistently ranked highest for maternal hemodynamic stability in low-risk parturients.

## Introduction and background

Cesarean delivery (CD) can be done with spinal anesthesia for its quick onset, profound analgesia, safety, and rapid return of potentials for speedy recovery [1,2]. However, despite the benefits, postspinal hypotension continues to occur frequently, up to 80%, and can cause symptoms of nausea, vomiting, and vertigo in the mother, as well as inadequate uteroplacental flow with implications for the welfare of the fetus [3-5]. Various methods to prevent and/or reduce postspinal hypotension have been advocated, and these include fluid preloading/coloading, leg compression, uterine displacement, reduction in the dose of local anesthetic, and the use of vasoactive drugs as adjunct agents [6]. Of these, the infusion of vasoactive drugs for the prevention of postspinal hypotension has been universally accepted and practiced as modern standard practice in obstetric anesthesia [7,8].

Of these, phenylephrine, norepinephrine, and ephedrine are the most commonly utilized vasopressors for this purpose [9,10]. The predominantly α1-adrenergic agonist phenylephrine is widely regarded as a first-line agent based on its potent vasoconstrictive properties and favorable neonatal acid-base profile relative to ephedrine [11]. Its administration, however, is often complicated by reflex maternal bradycardia and decreases in cardiac output [12]. Norepinephrine, with combined α-adrenergic and weak β-adrenergic activity, has emerged as a promising alternative that may provide improved heart-rate preservation and more stable maternal hemodynamics [13]. Ephedrine is an indirect α- and β-agonist characterized by a slower onset and longer duration of action compared to direct-acting agents. This limits its utility, but it does remain effective; however, with greater placental transfer compared with phenylephrine and norepinephrine, there is an associated increased risk of fetal acidosis and maternal side effects [14]. Despite the availability of these agents, significant heterogeneity persists in clinical practice, and the optimal prophylactic vasopressor, particularly for low-risk parturients, remains uncertain.

Previous systematic reviews and network meta-analyses of vasopressor efficacy in CD have largely included mixed or high-risk obstetric populations, limiting applicability to healthy women undergoing an elective procedure. Singh et al. performed a Bayesian network meta-analysis comparing vasopressors without stratifying outcomes by obstetric risk status [15]. Ryu et al. completed a similar comparison of multiple agents without distinguishing between risk categories [16]. Two more recent analyses compared norepinephrine against phenylephrine and reported equivalent efficacy for maintaining maternal blood pressure, with norepinephrine exhibiting a lower incidence of maternal bradycardia [17,18]. These collective observations highlight the need for a focused, comparative synthesis for low-risk elective CD.

The network meta-analysis offers a powerful approach to CIs combining direct and indirect information, especially when direct comparisons are unavailable, as in the case of obstructive anesthesia studies [19,20]. Maternal hemodynamic stability and neonatal outcomes such as Apgar scores, umbilical artery pH, and metabolic values are still pivotal to vasoactive drugs’ selection, apart from any other concern [21]. Though phenylephrine ensures optimal neonatal acid-base status, norepinephrine provides maternal hemodynamic benefits without harming the fetus, whereas ephedrine raises concerns about ensuring optimal A-B balance within the fetus in healthy term pregnancies compared to norepinephrine and phenylephrine, respectively [22-25]. Thus, we set a specific aim to systematically analyze the effectiveness and safety profile differences among the prophylactic uses of norepinephrine, phenylephrine, and ephedrine for preventing postspinal hypotension, as seen in low-risk pregnancies undergoing elective CD with spinal anesthesia.

## Review

Methodology

Data Sources and Search Strategy

A comprehensive literature search was performed in PubMed, Embase, and the Cochrane Central Register of Controlled Trials (CENTRAL). The search strategy used MeSH terms, Emtree terms, and free-text words related to cesarean section, spinal anesthesia, vasopressors, norepinephrine, phenylephrine, ephedrine, and prophylactic measures. Relevant reference lists in randomized controlled trials and previous systematic reviews were searched manually to look for more suitable trials. Only English-language publications were considered, as it would help in preventing biases in translated texts and ensure that the quality of reporting remains consistent across all trials. This systematic review was performed as per the Preferred Reporting Items for Systematic reviews and Meta-Analyses-Network Meta-Analysis (PRISMA-NMA) guidelines [20,26] and was registered in PROSPERO (CRD420251060720).

Study Selection

Trials published as randomized controlled studies between January 2015 and May 2025 were considered, as no studies were found to fit the inclusion criteria before 2015. The populations who could be included in studies were low-risk women undergoing elective cesarean section with spinal anesthesia. Studies with general anesthesia, emergencies, and other high-risk pregnancies such as preeclampsia and cardiac disease, as well as other than obstetrics, were excluded as they did not fit the homogeneous group. Trials were eligible if they examined the prophylactic use of norepinephrine, phenylephrine, or ephedrine, either together or in combination, given before or concurrent with spinal anesthesia, and in comparison to each other, placebo, or no treatment. The primary outcome was the rate of maternal hypotension. Secondary outcomes were maternal bradycardia, nausea and vomiting, and Apgar scores at one and five minutes. Records were exported into Rayyan QCRI for duplication removal and blinded screening [27]. Two reviewers (AB, NB) independently screened titles and abstracts, followed by full text-screening of potentially eligible studies. Any disagreements were resolved through discussion or referral to a third party (MH).

Data Extraction

Data were extracted independently by two researchers (AB and NB) using a standardized, pre-piloted data extraction form [28]. Data extracted included study and participant characteristics, intervention and drug details, and definitions of outcomes. If needed, primary authors were approached directly for clarification and/or additional data.

Risk of Bias and Quality Assessment

The risk of bias was judged using Cochrane Risk of Bias 2.0, assessing five domains (D1-D5). Two authors independently assessed each trial, and discrepancies were resolved through consensus. The risk of bias was judged to be low, some concerns, or high, and presented graphically. The quality of evidence for evidence certainty was assessed utilizing the GRADE approach. Evidence was graded as high (⊕⊕⊕⊕), moderate (⊕⊕⊕⊖), low (⊕⊕⊖⊖), and very low (⊕⊖⊖⊖), considering risk of bias, imprecision, and inconsistency [29].

Data Synthesis and Statistical Analysis

A frequentist random-effects network meta-analysis was performed to synthesize both direct and indirect comparisons among norepinephrine, phenylephrine, and ephedrine. For dichotomous outcomes, odds ratios (ORs) with 95% confidence intervals (CIs) were determined. Network geometry was displayed by using network plots, in which node size reflected the number of contributing studies and edge thickness represented the strength of direct evidence.

Treatment rankings were estimated using the surface under the cumulative ranking curve (SUCRA) values. Statistical analyses were conducted using STATA version 18.0 (StataCorp., College Station, TX, USA) and R version 4.3.2 (R Foundation, Vienna, Austria). Given anticipated clinical heterogeneity across trials (dose, timing, route of administration, and co-interventions), findings are interpreted cautiously. Transitivity was assessed qualitatively by comparing key clinical and methodological characteristics across comparisons. SUCRA rankings should be considered exploratory, and no causal treatment hierarchy was inferred.

Assessment of Publication Bias

Contour-enhanced funnel plots for postspinal hypotension and maternal bradycardia outcomes were created, with the help of significance contours with shades for p-values <0.01 and p-values <0.05. Egger’s regression test for the assessment of small study effects was employed. All analysis presented in Python code was done using Python 3.10, along with Matplotlib and NumPy for computation and plotting [30].

Sensitivity and Subgroup Analysis

The prespecified subgroup analyses included the administration method (bolus versus infusion), the type of comparator (placebo-controlled versus active-controlled), and geographic regions. These subgroup analyses are often useful in verifying consistency between the primary results and a set of preplanned subgroup comparisons.

Results

**Figure 1:**
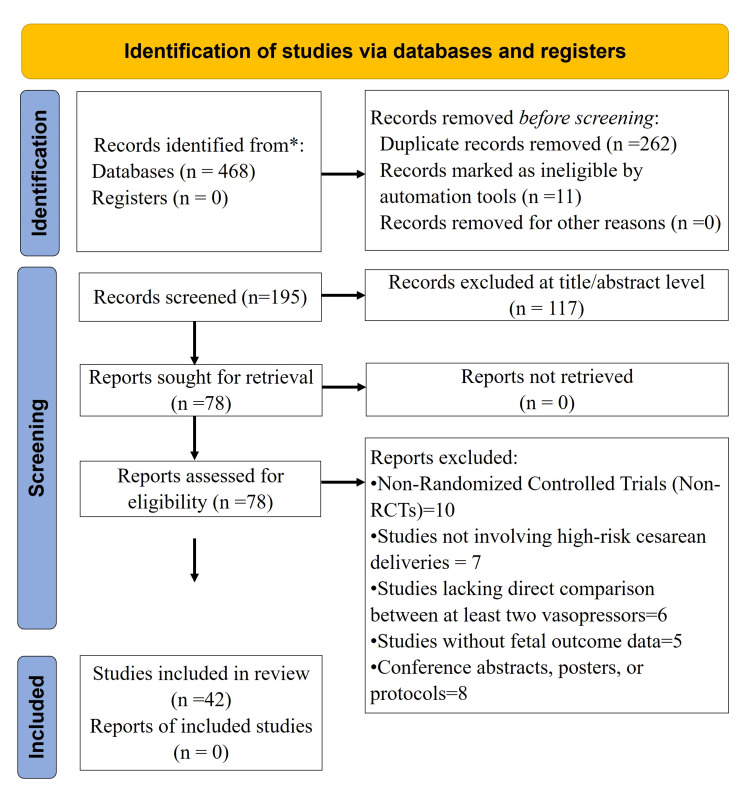
Preferred Reporting Items for Systematic reviews and Meta-Analyses (PRISMA) flowchart. *: Databases include PubMed, Embase, Cochrane Library, and Scopus.

Of the 468 records that were identified using PubMed, EMBASE, and Cochrane CENTRAL, 262 duplicates and 11 automatic exclusions were filtered out, leaving 195 records for screening the title/abstract. Overall, 117 were excluded as not relevant. Full texts of 78 studies were accessed; 36 were excluded, and 42 randomized controlled trials involving 4,534 patients were included (Figure 1). Studies spanned multiple regions and included low-risk term parturients undergoing elective CD under spinal anesthesia. Norepinephrine, phenylephrine, and ephedrine were the most widely studied vasopressors with varying protocols according to dosage, route, and adjuncts. The main outcomes were the incidence of postspinal hypotension and mean arterial pressure control. The secondary outcomes were bradycardia, nausea, vomiting, and neonatal parameters. Study characteristics are presented in Table 1.

**Table 1:** Study and patient characteristics of included studies. CD = cesarean delivery; ASA = American Society of Anesthesiologists; BE = base excess; BP = blood pressure; CO = cardiac output; CI = confidence interval/cardiac index; CSE = combined spinal-epidural; DIVA = difficult intravenous access; ED50 = effective dose for 50%; ED90 = effective dose for 90%; EP = ephedrine; HES = hydroxyethyl starch; HR = heart rate; HTN = hypertension; IV = intravenous; IVC = inferior vena cava; MAP = mean arterial pressure; MDPE = median performance error; MDAPE = median absolute performance error; ME = minimum effective; ME250 = minimum effective dose in 250 patients; MEV = minimum effective volume; NE = norepinephrine; ON = ondansetron; PE = phenylephrine; PI = perfusion index; SA = spinal anesthesia; SBP = systolic blood pressure; SVR = systemic vascular resistance; UA = umbilical artery; UV = umbilical vein; AU = Australia; CA = Canada; CN = China; EG = Egypt; HK = Hong Kong; IN = India; IR = Iran; PK = Pakistan; SG = Singapore; ZA = South Africa; UK = United Kingdom

Author/Reference	Year	Country	N	Population	Intervention	Comparator	Dose and route	Primary outcome	Secondary outcomes
Bishop et al. [31]	2017	ZA	506	Elective or urgent CD; SA	PE (bolus) vs. PE (infusion)	Bolus-only VP protocol	Infusion; fixed low-dose PE + rescue bolus	Severe PSH (MAP <70% baseline or SBP <80 mmHg)	HTN, complications
Buthelezi et al. [32]	2020	ZA	300	ASA I-II; elective CD; SA	PE (bolus) vs. PE (infusion + fluid coload)	PE bolus-only rescue	Infusion-like via fluid coload; 500 µg PE in 1 L	PSH (SBP <90 mmHg)	Bradycardia, Apgar, systolic HTN
Chen et al. [33]	2018	CN	120	ASA I-II; elective CD; SA	NE 5 vs. 10 vs. 15 µg/kg/h vs. Placebo	Saline infusion (placebo)	Infusion; 5-15 µg/kg/h	PSH	HTN, HR, BP stability, Apgar, blood gas
Chen et al. [34]	2020	CN	195	Elective CD; SA singleton	NE vs. Placebo	Saline (placebo)	Bolus + infusion	PSH	SBP stability, IVC-CI, bradycardia, Apgar, blood gas
Chen et al. [35]	2021	CN	99	ASA I-II; elective CD; SA	NE 0.025 vs. 0.05 vs. 0.075 vs. 0.1 µg/kg/min vs. Saline	Saline (placebo)	Infusion	PSH	SBP stability (MDPE, MDAPE), rescue NE, neonatal outcomes
Chen et al. [36]	2024	CN	23	Non-HTN term; CD; SA	NE with 0 vs. 5 vs. 10 mL/kg coload	Crystalloid coload volume (0 vs. 5 vs. 10 mL/kg)	Infusion, dose adjusted by up-down method	ED90 to prevent PSH	Dose reduction due to coload volume
Choudhary et al. [37]	2020	IN	90	Elective CD; SA	NE 6 µg vs. 4 µg vs. Placebo	Saline (placebo)	Bolus	PSH	Rescue NE requirement, Apgar, adverse effects
Elagamy et al. [38]	2021	EG	120	ASA II; elective CD; SA	NE vs. EP	EP 10 mg IV bolus	Bolus	PSH & doses needed	Nausea, vomiting, Apgar
Ali Elnabtity et al. [39]	2018	EG	122	Healthy term; elective CD SA	NE vs. EP	EP 10 mg IV bolus	Bolus; NE 5 µg vs EP 10 mg		Bradycardia, tachycardia, Apgar, uterine artery PI
Eskandr et al. [40]	2021	EG	75	Elective CD; SA	NE vs. EP vs. PE	Between-group (PE vs. NE vs. EP)	Infusion	PSH	HR, MAP, nausea, neonatal acidosis
Feng et al. [41]	2020	CN	238	Term; elective CD; SA	PE (0.25 µg/kg/min) vs. NE (0.05 µg/kg/min) vs. Placebo	NE (0.05 µg/kg/min) and placebo	Infusion	Maternal SBP and SVR under LiDCOrapid monitoring	UV/UA lactate, pH, incidence of nausea, bradycardia
Guo et al. [42]	2023	CN	80	Elective CD; SA	NE (bolus) vs. PE (bolus)	NE vs. PE bolus	Bolus; NE 8 µg vs PE 90 µg	ED90 and potency ratio	Bradycardia, Apgar, UA blood gas
Guo et al. [43]	2024	CN	175	Term; singleton; elective CD, SA	NE (0-0.1 µg/kg/min) + hydroxyethyl starch	NE dose groups: 0, 0.025, 0.05, 0.075, 0.1 µg/kg/min	Infusion with colloid coload	PSH	ED90, SBP stability, Apgar, UA blood gas
Hasanin et al. [44]	2019	EG	123	Elective CD; SA	NE (0.05 µg/kg/min) vs. PE (0.75 µg/kg/min)	PE infusion (0.75 µg/kg/min)	Infusion, adjusted manually	PSH	Bradycardia, reactive HTN, neonatal outcomes
Hasanin et al. [45]	2019a	EG	284	Term; elective CD; SA	NE 0.025 vs. 0.05 vs. 0.075 µg/kg/min	Dose comparison	Infusion	PSH	SBP, HR, HTN, bradycardia, neonatal outcomes
Hasanin et al. [46]	2019b	EG	217	Elective CD; SA	PE bolus vs. fixed infusion vs. variable infusion	Different PE regimens	Bolus vs. fixed vs. variable infusion	MAP	Bradycardia, reactive HTN, physician interventions
Hassabelnaby et al. [47]	2024	EG	196	Term; elective CD; SA	Epinephrine (0.03 µg/kg/min) vs. PE (0.4 µg/kg/min)	PE 0.4 µg/kg/min infusion	Infusion	Composite of hypo/HTN, brady/tachycardia	Apgar, UA blood gas
Hiruthick et al. [48]	2021	IN	120	ASA I-II; elective CD; SA	PE 75 µg vs. 100 µg	PE 100 µg bolus	Bolus	PSH	Bradycardia, BP, Apgar
Hosny et al. [49]	2020	EG	100	ASA I-II; elective CD; SA	PE 100 µg vs. Placebo	Saline (placebo)	Bolus	PSH	Apgar, BE, HR, BP
Jaitawat et al. [50]	2019	IN	120	Elective CD’ SA	PE 75 µg vs. 100 µg vs. Placebo	PE 100 µg bolus and placebo	Bolus	PSH	Bradycardia, rescue use, Apgar
Lashari et al. [51]	2021	PK	90	Elective CD; SA	EP 10 mg vs. Saline	Saline (placebo)	Bolus; 10 mg IV	PSH	BP trends, procedural safety
Loubert et al. [52]	2017	CA	30	Healthy; term; elective CD; SA	PE infusion with variable fluid volume (colloid preload)	Variable HES preload volume (500–900 mL)	Colloid preload + PE infusion	Minimum effective volume (MEV) to prevent PSH	Cardiac output, stroke volume
McDonnell et al. [53]	2017	AU/UK	185	Elective CD; SA or CSE	PE infusion vs. ME infusion	ME250 µg/min infusion	Infusion	UA arterial pH	Maternal hemodynamics, neonatal outcomes
Mon et al. [54]	2017	UK	40	Elective CD; SA	PE 100 µg/min vs. EP 5 mg/min	EP 5 mg/min infusion	Infusion	CO	UA pH, SBP, HR, fetal acidosis
Moslemi et al. [55]	2015	IR	90	Elective CD; SA	PE infusion vs. EP infusion vs. Placebo	Saline (placebo)	Infusion	PSH	Apgar, fetal acidosis, maternal complications
Ngan Kee et al. [56]	2020	HK	668	Elective and non-elective CD; SA or CSE	NE infusion vs. PE infusion	PE 100 µg/mL infusion	Infusion	UA pH	Base excess, hypotension episodes
Nikooseresht et al. [57]	2020	IR	120	Healthy; elective CD; SA	PE 100 µg bolus vs. 50 µg/min infusion vs. Placebo	Saline (placebo)	Bolus or Infusion	PSH	Rescue PE, HR, Apgar, nausea, BP trends
Qian et al. [58]	2020	CN	75	Elective CD; CSE	PE infusion adjusted by ON timing	Timing of ON	Infusion	ED50 of PE	Dose adjustment by ON timing
Qin et al. [59]	2022	CN	193	Elective CD; ≥37 weeks	Crystalloid preload (0, 4, 8, 12 mL/kg) + NE infusion	Crystalloid preload (0, 4, 8, 12 mL/kg)	Bolus + Infusion	IVC collapsibility index & PSH	BP, IVC changes, SBP, CI trend
Rana et al. [60]	2020	PK	80	Elective CD; SA	PE variable infusion + rescue boluses vs. Rescue boluses alone	PE rescue bolus only	Infusion + bolus	PSH	Maternal SBP, drug efficacy
Shafeinia et al. [61]	2020	IR	116	Elective CD; SA	NE variable-rate infusion vs. fixed-rate infusion	Saline (placebo)	Infusion	Maternal hemodynamic changes	Apgar, UA pH, nausea, vomiting
Shen et al. [62]	2022	CN	161	Elective CD; SA	NE 0.02-0.06 µg/kg/min (dose-response in singleton vs. twin)	Fixed-rate infusion	Infusion	MAP prevention	Bradycardia, , reactive HTN
Sheng et al. [63]	2021	CN	237	Singleton vs twin; CD, SA	NE infusion (0.02-0.10 µg/kg/min) + ON vs. Placebo	Placebo	Infusion	PSH	Reactive HTN, bradycardia, neonatal outcomes
Sheng et al. [64]	2024	CN	150	Elective CD, SA	NE infusion (0.02-0.10 µg/kg/min) + ON vs. Placebo	Saline (Placebo)	Infusion	NE requirement	Apgar, bradycardia, nausea
Singh et al. [65]	2022	IN	60	Singleton; term; elective CD, SA	NE 5 µg/min vs. PE 100 µg/min	None	Infusion	UA pH	Bradycardia, VP use, neonatal outcomes
Singh et al. [66]	2022a	IN	60	Elective CD; SA	NE 2.5 µg/min vs. PE 50 µg/min	NE infusion	Infusion	UA pH	Bradycardia, Apgar, MAP
Sun et al. [67]	2024	CN	223	Singleton; CD, SA	NE 0.08 µg/kg/min vs. PE 0.5 µg/kg/min	None	Infusion	Fetal HR & CO	Bradycardia, MAP, Apgar
Tan et al. [68]	2023	SG	94	Elective CD, SA	ADIVA system (PE/EP) vs. DIVA system (PE/EP)	Standard of care	Infusion	PSH	VP use, UA pH, neonatal outcomes
Wang et al. [69]	2020	CN	82	Elective CD; SA	Epinephrine 0.1 µg/kg/min vs. PE 1 µg/kg/min	PE infusion	Infusion	MAP, HR, CO	UA pH, bradycardia, VP boluses
Wei et al. [70]	2020	CN	99	Elective CD; SA	NE infusion: 0.04, 0.05, 0.06, 0.07 mg/kg/min vs. Placebo	None	Infusion	PSH	Apgar, UA pH, bradycardia
Xu et al. [71]	2019	CN	97	Elective CD; SA	NE 4 mg/min vs. EP 4 mg/min (infusion)	EP 4 mg/min	Infusion	Tachycardia	Bradycardia, HTN, maternal hemodynamics, neonatal outcomes
Xu et al. [72]	2021	CN	100	Elective CD; CSE	NE infusion: 0.025, 0.05, 0.075, 0.1 µg/kg/min vs. Placebo	None	Infusion	Dose-response; PDH prevention	Apgar, UA pH, physician intervention rates

Risk of Bias Assessment

**Figure 2:**
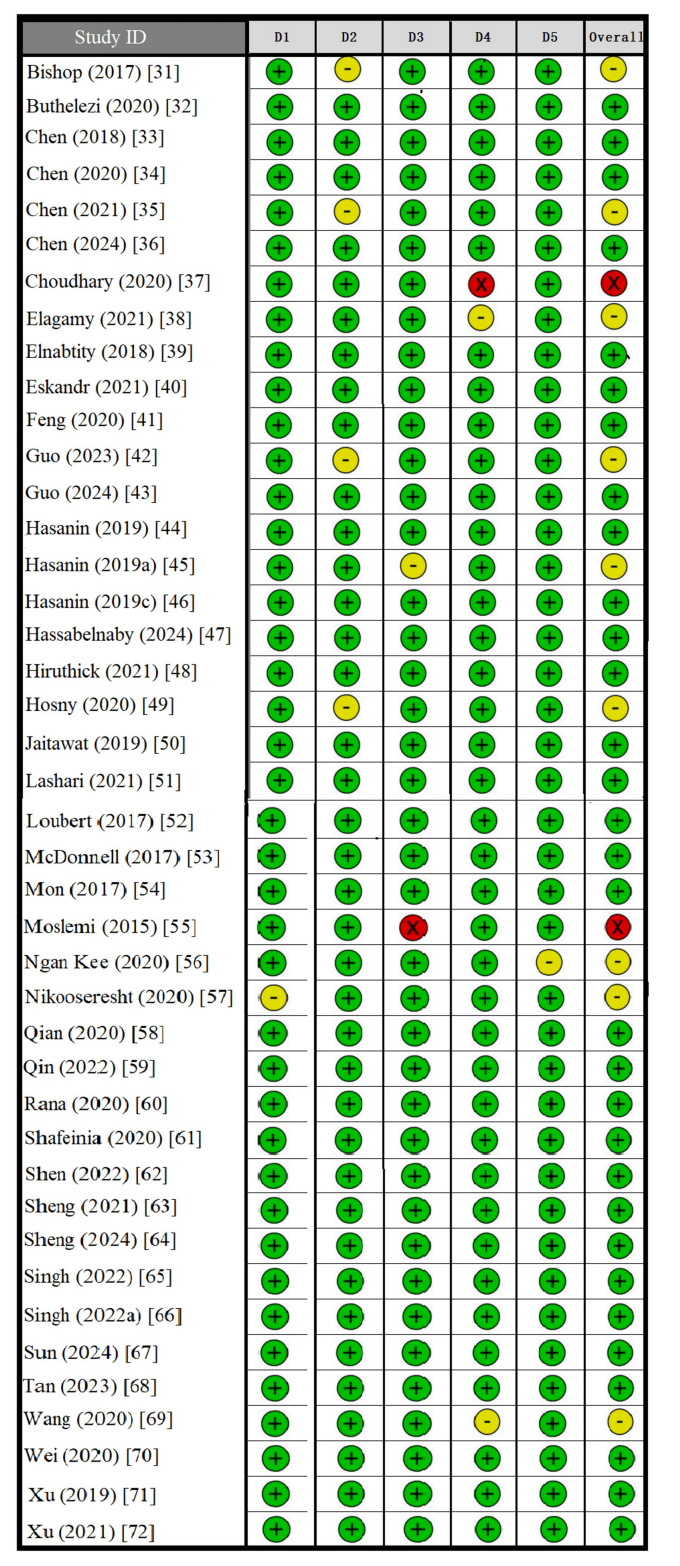
Risk of bias summary plot for included studies assessed by the Cochrane Risk of Bias Tool (RoB 2). D1: Bias arising from the randomization process. D2: Bias due to deviations from intended interventions. D3: Bias due to missing outcome data. D4: Bias in the measurement of the outcome. D5: Bias in the selection of the reported result.

Most studies had a low risk of bias across all domains, and 33 trials were judged to be low risk overall. There were some trials with “concerns” primarily in D2 and D5, with minor issues in intervention adherence or selective reporting. Two studies were judged to be at high risk because they had serious concerns regarding outcome measurement (D4) and reporting bias (D5) [37,55]. Despite these exceptions, the overall methodological quality of the included studies was deemed strong, offering confidence in the reliability of the network meta-analysis findings (Figure 2).

Outcomes

**Figure 3:**
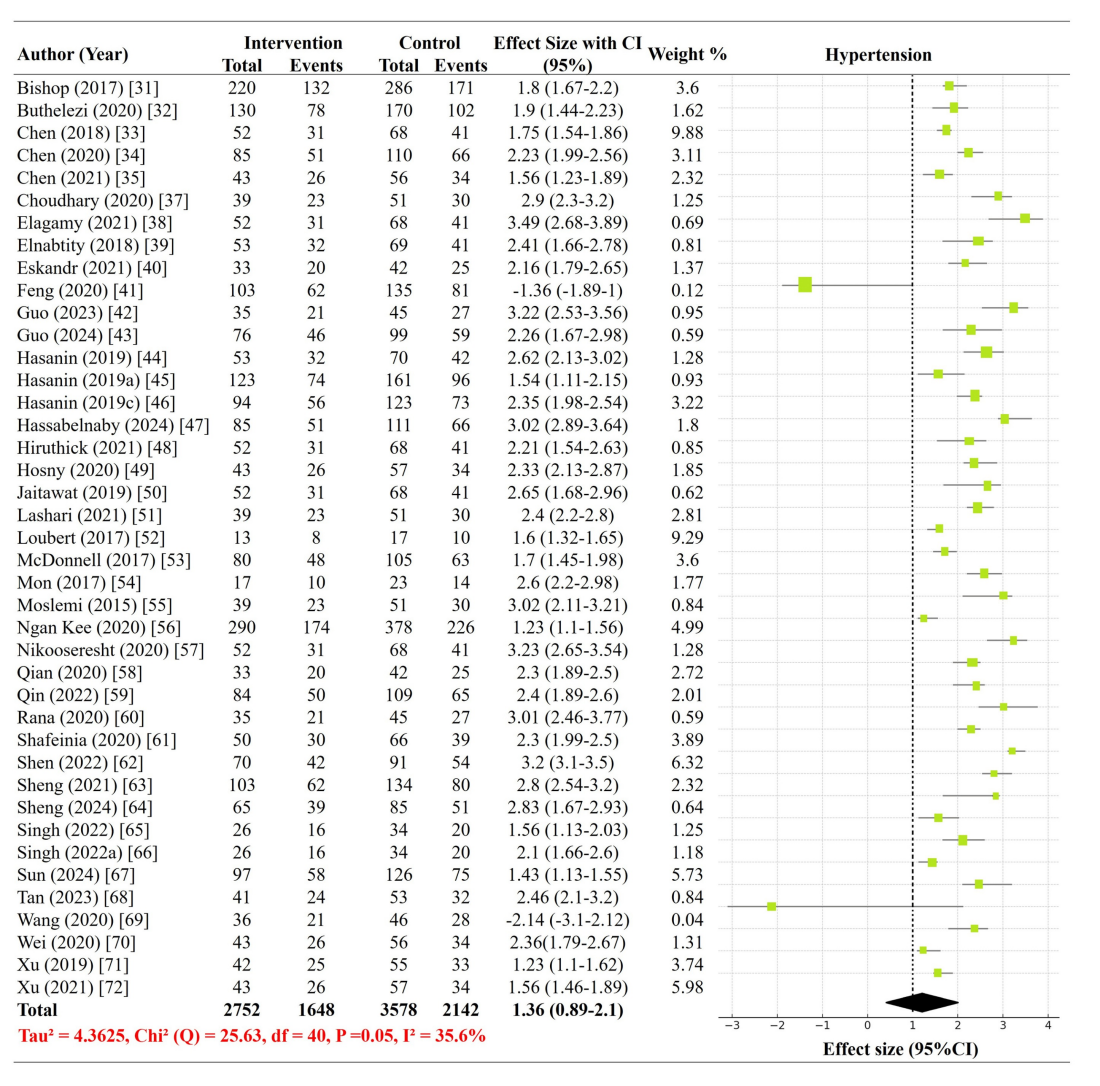
Forest plot of the incidence of postspinal hypotension with vasopressor use during elective cesarean delivery under neuraxial anesthesia.

Incidence of hypotension: Of the 42 included trials, 894 received phenylephrine, 391 received norepinephrine, 69 received ephedrine, and 451 received placebo for the primary outcome, reflecting the relative frequency with which these agents have been evaluated for the management of postspinal hypotension during CD. Importantly, the comparator arms varied across studies, ranging from placebo or saline to other active vasopressors, which may have influenced the magnitude of the pooled treatment effects. The overall pooled effect size was 1.36 (95% CI = 0.89-2.10), indicating a non-significant reduction in postspinal hypotension risk among vasopressor recipients compared to controls, though this result should be interpreted with caution given the heterogeneity in control interventions. Although some individual trials reported significant effects, pooled estimates showed overlapping CIs, indicating no clear superiority at the aggregate level, including those by Hasanin et al. (2019), Chen et al. (2021), and Singh et al. (2022), where CIs did not cross the line of no effect [35,44,65]. However, there was moderate between-study heterogeneity (τ = 4.3625, I² = 35.6%, p = 0.05), which was likely attributable to differences in vasopressor type, dosing regimens, and administration routes. These findings emphasize the complex and context-dependent impact of vasopressor protocols and reinforce the importance of tailoring vasopressor selection to individual maternal and procedural characteristics (Figure 3).

**Figure 4:**
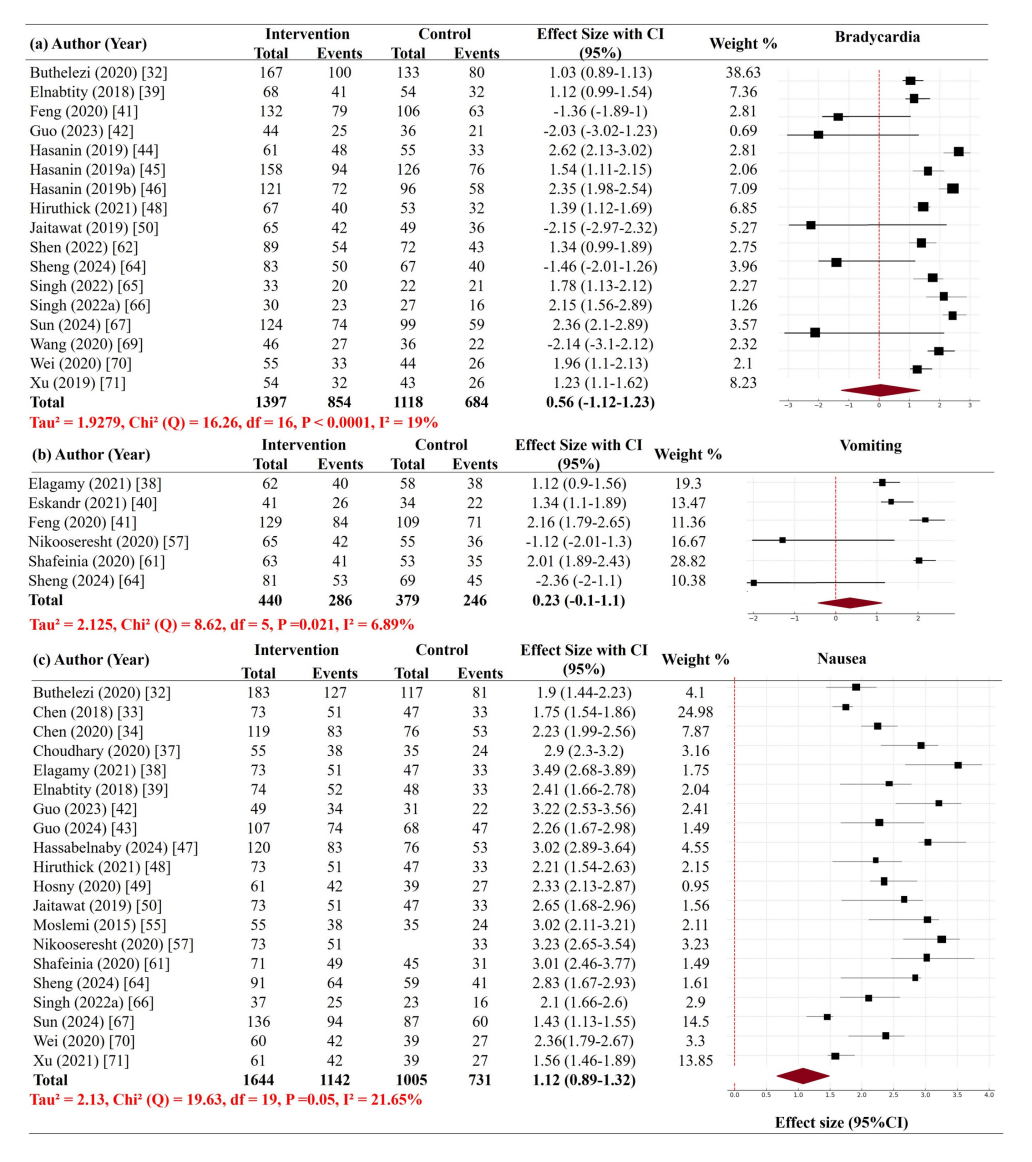
Forest plot of secondary outcome measures. (a) The incidence of maternal bradycardia. (b) The incidence of nausea and vomiting. (c) Neonatal Apgar scores at one and five minutes.

Incidence of bradycardia: The pooled analysis of maternal bradycardia incidence included 1,397 patients in the intervention arms and 1,118 patients in the control arms from 17 trials [32,39,41,42, 44-46,50,62,64-67,69-71]. The overall effect was -0.56 (95% CI = -1.12-1.23), suggesting a non-significant trend toward reduced bradycardia with vasopressor intervention. Heterogeneity was low (I² = 19%, p < 0.0001), indicating consistency of results across studies. However, some studies, such as Hasanin et al. (2019) and Sun et al. (2024), reported strongly positive outcomes in intervention groups with signs of potential agent-specific effects [44-46,67] (Figure 4a).

Incidence of nausea and vomiting: Six trials [24,38,41,57,61,64] investigated maternal nausea and vomiting, involving 400 intervention group patients and 379 control group patients. The overall effect size was 0.23 (95% CI = -0.1-1.1), with no difference between groups. A moderate degree of heterogeneity was observed (I² = 6.89%, p = 0.021). While individual trials such as Feng et al. and Shafeinia et al. (2020) suggested a reduction in nausea and vomiting with some vasopressors, the pooled result did not reach statistical significance [41,61] (Figure 4b).

Neonatal Apgar scores at one and five minutes: A meta-analysis was conducted for Apgar scores in 20 trials [32-34,37-39,42,43,47-50,55,57,61,64,65,67,70,72], out of which 1,644 neonates were in the intervention group and 1,005 in the control group. The overall effect size was pooled to 1.12 (95% CI = 0.89-1.32), with no significant difference between groups. The heterogeneity was low (I² = 21.65%, p = 0.05). Surprisingly, studies by Moslemi and Rasooli (2015), Nikooseresht et al. (2020), and Sheng et al. (2024) demonstrated a notable improvement in Apgar scores favoring intervention groups, suggesting that certain vasopressor interventions may be associated with better neonatal outcomes [55,57,64] (Figure 4c).

Neonatal Acid-Base Status

Eleven studies reported umbilical artery pH or base excess as indicators of neonatal acid-base balance. Among these, most studies demonstrated no significant differences between vasopressors. For instance, Singh et al. (2022a) and Ngan Kee (2020) reported comparable mean umbilical artery pH between the norepinephrine and phenylephrine groups (p > 0.05), while Moslemi and Rasooli (2015) observed a slightly higher incidence of fetal acidosis in the ephedrine group. Guo et al. (2023) and Feng et al. (2020) also found no statistically significant difference in base excess values between norepinephrine and phenylephrine, although ephedrine trended toward lower pH. Owing to the heterogeneity in reporting metrics (some as continuous pH values, others as % acidosis), a pooled meta-analysis was not feasible. However, the available data collectively suggest that norepinephrine and phenylephrine are comparable in terms of neonatal acid-base status, whereas ephedrine may be associated with a higher risk of acidosis (Table 1).

SUCRA Scores for Various Vasopressors

Based on the network meta-analysis, the SUCRA scores for various vasopressors in preventing postspinal hypotension and maternal bradycardia during CD under spinal anesthesia were determined. These findings indicate that norepinephrine has the highest probability of being the most effective vasopressor for preventing hypotension (92%) and minimizing the risk of bradycardia (88%). Phenylephrine showed moderate efficacy (71% preventing hypotension and 44% minimizing the risk of bradycardia), whereas ephedrine ranked the lowest among the three by preventing hypotension by 32% and minimizing the risk of bradycardia by 19%. These results align with those of Liu et al. (2022) and Kang et al. (2024), suggesting superior hemodynamic stability provided by norepinephrine during spinal anesthesia for CD in low-risk parturients. In summary, SUCRA analysis suggested a higher probability of favorable outcomes with norepinephrine compared with phenylephrine and ephedrine; however, these rankings should be interpreted as exploratory and not definitive evidence of superiority.

Network Meta-Analysis

**Figure 5:**
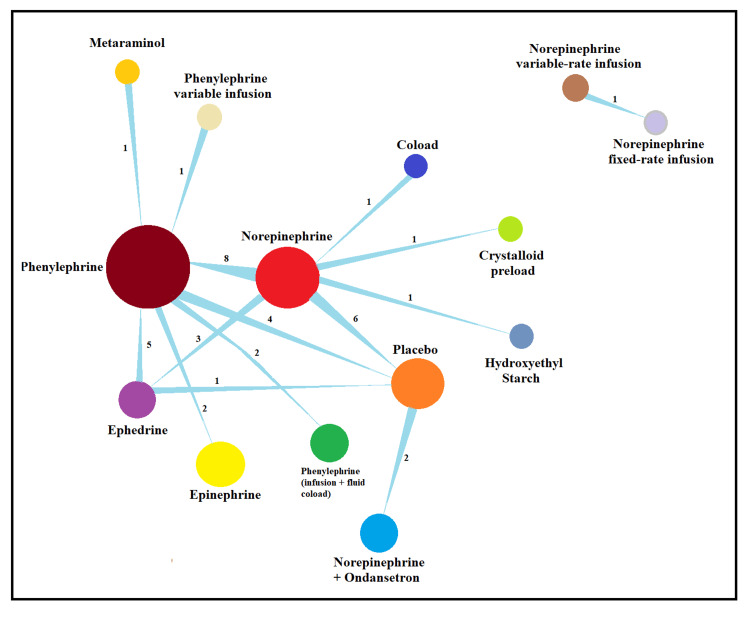
Network diagram of treatment comparisons for the primary outcome (postspinal hypotension).

Phenylephrine and norepinephrine served as central nodes in the treatment network. Phenylephrine, the most frequently studied vasopressor, was directly compared with norepinephrine (eight trials), ephedrine (five trials), epinephrine (one trial), and placebo (six trials), as well as in combination regimens (e.g., fluid coloading, variable-rate infusion). Norepinephrine, the second most interconnected agent, was compared with phenylephrine, placebo, ephedrine, and in combinations (e.g., norepinephrine plus ondansetron, fixed- vs. variable-rate infusion). Although agents such as metaraminol and epinephrine appeared in some trials, they were only included if comparisons involved phenylephrine, norepinephrine, or ephedrine. The network diagram shows a well-connected structure, supporting robust comparisons (Figure 5).

Publication Bias

**Figure 6:**
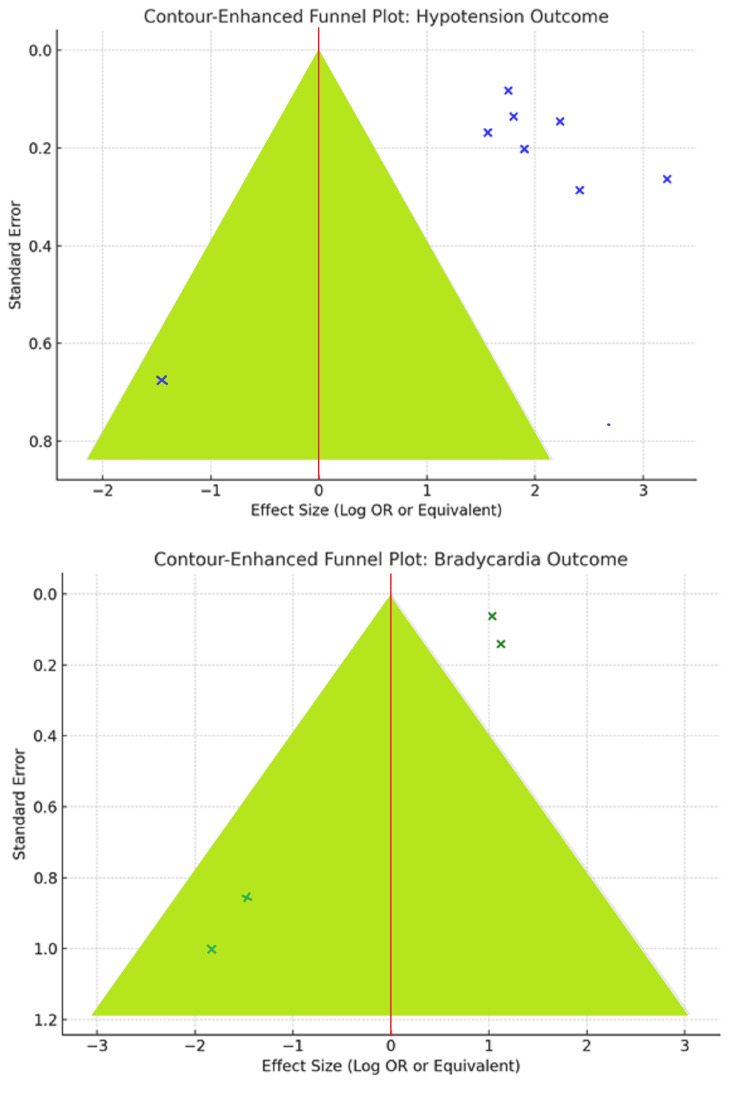
Contour-enhanced funnel plot for postspinal hypotension and bradycardia.

To assess potential small-study effects, contour-enhanced funnel plots for hypotension and bradycardia outcomes were plotted. These plots allow the differentiation between asymmetry due to publication bias and heterogeneity. On visual inspection, there was a symmetrical distribution of studies around the effect size with no bunching in non-significant regions (p > 0.05). Shading based on p-value cut-offs also showed balanced reporting of significant and non-significant results. Overall, the symmetrical shapes suggest minimal publication bias and support the validity of SUCRA rankings and conclusions drawn from this network meta-analysis (Figure 6).

Subgroup Analysis

Subgroup analysis examined sources of heterogeneity and consistency of vasopressor efficacy by country, comparing agent, and route of administration. Pakistani trials showed the strongest effect (OR = 2.29, 95% CI = 1.52-2.51), followed by India (OR = 2.45, 95% CI = 0.78-7.77), but the latter was with high heterogeneity (I² = 63.01%). Among vasopressor comparisons, phenylephrine versus ephedrine (OR = 2.19, 95% CI = 1.32-2.31, p = 0.011) and norepinephrine versus ephedrine (OR = 1.89, 95% CI = 1.27-2.26, p < 0.0001) significantly prevented postspinal hypotension. Phenylephrine bolus versus infusion + fluid coload was also effective (OR = 2.04, 95% CI = 1.42-2.41, I² = 8.27%). Bolus dosing (OR = 1.99, 95% CI = 1.47-2.46) and fixed versus variable infusion (OR = 1.54, 95% CI = 1.08-2.07) were effective with moderate heterogeneity. However, phenylephrine infusion with colloid preload was not significant (OR = 0.56, 95% CI = 0.21-1.10). The findings demonstrate regional variation, optimal vasopressor combinations, and administration protocols for maximizing postspinal hypotension prevention in low-risk CDs (Table 2).

**Table 2:** Subgroup analysis by country, vasopressor comparison and route (bolus vs. infusion). CP = crystalloid preload; NE = norepinephrine; EP = ephedrine; PE = phenylephrine; E = epinephrine; ON = ondansetron; HES = hydroxyethyl starch; AU = Australia; CA = Canada; CN = China; EG = Egypt; HK = Hong Kong; IN = India; IR = Iran; PK = Pakistan; SG = Singapore; ZA = South Africa; UK = United Kingdom

Variables	Subgroups	Number of studies	Sample size	Effect Size with 95% CI	P-value	Heterogeneity: I² (%)
Country	AU/UK [53]	1	185	1.58 (1.02–2.01)	0.51	29.21
	CA [52]	1	30	1.57 (0.92–1.91)	0.21	8.91
	CN [33-36,41-43,58,59,62-64,67,69-72]	17	2,324	1.59 (1.02–2.01)	0.06	14.11
	EG [38-40,44-47,49]	8	1,237	1.61 (0.97–1.96)	0.001	24.31
	HK [56]	1	668	1.51 (0.86–1.85)	0.16	17.02
	IN [37,48,50,65,66]	5	450	1.45 (0.78–1.77)	0.71	19.11
	IR [55,57,61]	3	326	0.56 (0.21–1.20)	0.51	26.3
	PK [51,60]	2	170	1.61 (0.92–1.91)	0.21	44.31
	SG [68]	1	94	1.44 (0.82–1.91)	0.26	9.11
	ZA [31,32]	2	806	1.57 (0.94–1.93)	0.05	13.81
	UK [54]	1	40	1.49 (0.87–1.86)	0.16	19.11
VP compared	CP vs. NE [59]	1	238	1.63 (1.04–2.03)	0.71	49.01
	EP vs. saline [51]	1	100	1.54 (0.97–1.96)	0.06	19.11
	E vs. PE [47,69]	2	275	1.47 (0.86–1.85)	0.05	23.91
	NE + ON vs. placebo [63,64]	2	160	1.46 (0.84–1.83)	0.03	39.31
	NE variable-rate infusion vs. NE fixed-rate infusion [61]	1	120	1.50 (0.92–1.91)	0.06	34.01
	NE vs. HES [43]	1	60	1.55 (0.93–1.92)	0.31	44.02
	NE vs. coload [36]	1	123	1.89 (1.12–2.11)	0.04	29.01
	NE vs. EP [38,39,71]	3	321	1.99 (1.22–2.21)	0.13	24.11
	NE vs. EP vs. PE [40]	1	284	1.69 (0.92–1.91)	0.02	28.91
	NE vs. PE [41,42,44,56,62,65-67]	8	967	1.59 (1.12–2.11)	0.03	9.01
	NE vs. placebo [33,34,37,70,72]	5	573	2.19 (1.32–2.31)	0.011	34.01
	PE vs. PE variable infusion [46]	1	120	1.94 (1.21–2.20)	0.01	19.11
	PE vs. placebo [48,49,50,57]	4	1,519	1.71 (1.05–2.04)	0.05	19.31
	PE vs. EP [54,55]	2	90	1.89 (1.27–2.26)	0.0001	13.27
	PE vs. metaraminol [53]	1	116	2.29 (1.52–2.51)	0.05	23.27
Dose and route	Bolus [37-39,42,48-51]	8	842	1.99 (1.47–2.46)	0.001	25.72
	Bolus + infusion [34,46,57,59,60]	5	1,094	1.54 (1.08–2.07)	0.01	19.05
	Bolus vs. fixed vs. variable infusion [31]	1	217	1.32 (0.77–1.76)	0.05	30.94
	CP + PE infusion [32,43]	2	205	1.68 (1.22–2.21)	0.03	27.05
	Infusion [33,35,36,40,41,44,45,47,53-56,58,61-72]	26	3,927	0.56 (0.21–1.20)	0.001	8.6

Discussion

Maternal hypotension in the setting of spinal anesthesia for the induction of elective cesarean sections has long remained a significant and practical concern, with established implications for both mothers and their newborns in obstetric literature [17,50,53,62,68]. The aim of this network meta-analysis is, in fact, a fulfillment of a particular need in this literature, as we were able to perform a comparative analysis concerning the preventive use of vasopressors in low-risk partrients in the process of elective cesarean sections with spinal anesthesia, in contrast with previously published meta-analytic literature compilations that covered a broad heterogeneous range of conditions and cases in a less precise manner [2,4,7,10,12,25,35,50,53,55].

While prior meta-analyses have demonstrated norepinephrine and phenylephrine to be hemodynamically desirable in comparison to other agents, they were predominantly conducted in mixed-risk patients and for non-prophylactic or procedural purposes in emergency settings. Ryu et al. outlined prophylactic advantages of norepinephrine and mephentermine when administered by infusion techniques, but were constrained by their focus on high-risk pregnancies and non-prophylactic regimens [59,73]. This was also true for the Bayesian network meta-analysis conducted by Singh et al., where maternal and fetal outcomes were assessed and compared between treatment regimens but lacked stratification for maternal or fetal risks of delivery or for elective versus non-elective procedures in healthy term pregnancies [15]. Subsequent analyses were more recent studies performed by Liu et al. and Kang et al., both of which showed norepinephrine to decrease maternal bradycardia with no change in neonatal outcomes, but were conducted in mixed elective and non-elective scenarios [17,18].

By narrowing the inclusion criteria for analysis to that of randomized controlled trials assessing the use of prophylactic vasopressor infusions in low-risk elective cesarean sections, the results enhance internal validity and relevance. For the primary outcome measure of postspinal hypotension in 42 trials and 4,534 women, no significant differences were found pairwise between phenylephrine, norepinephrine, and ephedrine. However, the results of the SUCRA analysis consistently placed a superiority ranking upon norepinephrine, followed by phenylephrine and ephedrine, for both the prevention of hypotension and the maintenance of maternal heart rate.

For the secondary outcomes, norepinephrine had a decreased incidence of maternal bradycardia and nausea, as expected [17,18]. Notably, the maternal outcomes in the norepinephrine group had an unrelated adverse neonatal outcome in that the Apgar scores and cord blood analyses both showed the agents to be equivalent. Phenylephrine was effective for blood pressure but had a significant risk for reflex bradycardia and possible cardiac output decrease. Even in a low-risk population, this could have significant clinical relevance. Ephedrine was consistently the least effective and had risks associated with fetal acidosis, an unstable blood pressure response, and high metabolic risks for the mother, all of which have been reported in previous studies [15,65,66,73-75].

Some limitations are worth mentioning. The reporting of secondary outcomes, including nausea, vomiting, reactive hypertension, and rescue vasopressor, was not homogeneous, which made a quantitative analysis challenging. Dose, time, and route of administration of vasopressors introduced clinical diversity. Although a visual inspection for transitivity and inconsistency and a further examination for the plausible presence of transitivity and inconsistency showed no major points for concern, the lack of direct comparisons and trial sequential analysis leaves a concern for the confidence in cumulative evidence accumulation. The presence of publication bias was identified upon inspection and Egger’s test, which is a known problem in the literature in obstetric anesthesiology. Despite these limitations, a large study population, careful study population selection, and a network analysis increased the confidence in our results.

In summary, this network meta-analysis provides the most focused evidence to date on prophylactic vasopressor use for postspinal hypotension in low-risk elective CD. While no agent demonstrated clear statistical superiority, norepinephrine consistently ranked highest for maternal hemodynamic stability and bradycardia prevention without compromising neonatal safety. These findings support norepinephrine as a preferred prophylactic option in this population and highlight the need for future large-scale, population-specific trials to establish definitive clinical guidelines.

## Conclusions

This network meta-analysis provides the most current and focused synthesis of evidence on prophylactic vasopressor use for postspinal hypotension in low-risk parturients undergoing elective CD under spinal anesthesia. Based on 42 randomized controlled trials, our analysis found no statistically significant differences among phenylephrine, norepinephrine, and ephedrine for postspinal hypotension prevention; however, SUCRA rankings consistently favored norepinephrine for better maternal hemodynamic stability and lower bradycardia incidence. Norepinephrine maintained neonatal safety, while ephedrine showed poorer outcomes. Across randomized trials in low-risk elective CD, no vasopressor demonstrated statistically significant superiority for the prevention of postspinal hypotension. Exploratory ranking analyses consistently favored norepinephrine for maternal hemodynamic stability, but these findings should be interpreted cautiously given clinical heterogeneity and inconsistent outcome definitions. Definitive recommendations require harmonized outcome reporting and adequately powered head-to-head trials.

## Appendices

**Table 3:** Summary of study selection process for inclusion in the systematic review and network meta-analysis.

Source	Search string
PubMed	(“Cesarean Section”[Mesh] OR “cesarean section”[tiab] OR “cesarean delivery”[tiab] OR “caesarean section”[tiab] OR “caesarean delivery”[tiab]) AND (“Norepinephrine”[Mesh] OR “norepinephrine”[tiab] OR “noradrenaline”[tiab]) AND (“Anesthesia, Spinal”[Mesh] OR “Anesthesia, Epidural”[Mesh] OR “Anesthesia, Neuraxial”[Mesh] OR “spinal anesthesia”[tiab] OR “neuraxial anesthesia”[tiab] OR “epidural anesthesia”[tiab] OR “combined spinal epidural”[tiab]) AND (“elective”[tiab] OR “planned”[tiab])
Cochrane Library	(caesarean OR cesarean OR “C-section” OR “caesarean delivery” OR “cesarean delivery”) AND (norepinephrine OR noradrenaline) AND (“spinal anesthesia” OR “neuraxial anesthesia” OR “epidural anesthesia” OR “combined spinal epidural”) AND (elective OR planned)
Embase	(‘cesarean section’/exp OR ‘cesarean section’:ti,ab OR ‘cesarean delivery’:ti,ab OR ‘caesarean section’:ti,ab OR ‘caesarean delivery’:ti,ab) AND (‘norepinephrine’/exp OR ‘norepinephrine’:ti,ab OR ‘noradrenaline’:ti,ab) AND (‘spinal anesthesia’/exp OR ‘epidural anesthesia’/exp OR ‘neuraxial anesthesia’/exp OR ‘spinal anesthesia’:ti,ab OR ‘neuraxial anesthesia’:ti,ab OR ‘epidural anesthesia’:ti,ab OR ‘combined spinal epidural’:ti,ab) AND (‘elective’:ti,ab OR ‘planned’:ti,ab)
